# Sex difference in association of symptoms and white matter deficits in first-episode and drug-naive schizophrenia

**DOI:** 10.1038/s41398-018-0346-9

**Published:** 2018-12-18

**Authors:** Xiao-E Lang, Daomin Zhu, Guangya Zhang, Xiangdong Du, Qiufang Jia, Guangzhong Yin, Dachun Chen, Meihong Xiu, Bo Cao, Li Wang, Xiaosi Li, Jair C. Soares, Xiang Yang Zhang

**Affiliations:** 1 Department of Psychiatry, The First Clinical Medical College, Shanxi Medical University, Taiyuan, Shanxi Province China; 2 Hefei Fourth People’s Hospital, Anhui Mental Health Center, Hefei, China; 3 Suzhou Psychiatric Hospital, The Affiliated Guangji Hospital of Soochow University, Jiangsu, China; 4 Psychiatry Research Center, Beijing HuiLongGuan Hospital, Peking University, Beijing, China; 5 Department of Psychiatry and Behavioral Sciences, The University of Texas Health Science Center at Houston, Houston, Texas USA; 6 CAS Key Laboratory of Mental Health, Institute of Psychology, Chinese Academy of Sciences, Beijing, China

## Abstract

Accumulating evidence shows that disruption of white matter (WM) may be involved in the pathophysiology of schizophrenia, even at the onset of psychosis. However, very few studies have explored sex difference in its association with psychopathology in schizophrenia. This study aims to compare sex differences in clinical features and WM abnormalities in first-episode and drug-naive (FEDN) schizophrenia among Han Chinese inpatients. The WM fractional anisotropy (FA) values of the whole-brain were determined using voxel-based diffusion tensor imaging (DTI) in 39 (16 males and 23 females) FEDN patients with schizophrenia and 30 healthy controls (13 males and 17 females) matched for gender, age, and education. Patient psychopathology was assessed using the Positive and Negative Syndrome Scale (PANSS).Our results showed that compared with the controls, the patients showed widespread areas of lower FA, including corpus callosum, brainstem, internal capsule, cingulate, and cerebellum (all adjusted *p* < 0.01). Further, male patients showed lower FA values in left cingulate (*F* = 4.92, *p* = 0.033), but higher scores on the PANSS total, positive, and general psychopathology subscale scores (all *p* < 0.01) than female patients. Multivariate regression analysis showed that for male patients, FA values in right corpus callosum were positively associated with the PANSS total (beta = 0.785, *t* = 3.76, *p* = 0.002) and the negative symptom scores (beta = 0.494, *t* = 2.20, *p* = 0.044), while for female patients, FA values in left cingulate were negatively associated with the PANSS positive symptom score (beta = −0.717, *t* = −2.25, *p* = 0.041). Our findings indicate sex difference in white matter disconnectivity and its association with psychopathological symptoms in an early course of schizophrenia onset.

## Introduction

Increasing evidence has consistently displayed sex differences in many aspects of schizophrenia^1–5^. For example, male patients show earlier age of onset, more severe symptoms, worse cognitive function, poorer treatment response, and worse global outcome than female patients^6–14^. Moreover, male patients have less education, have held jobs less often, and function worse socially than females^6,7,10,15^. However, the pathophysiological mechanisms underlying these sex differences in schizophrenia patients are still unknown.

The disconnection within and between brain regions has long been proposed to explain the brain pathology associated with schizophrenia^16^. Gray matter regions (“neurons”) are connected by white matter (WM) (“axons”; fiber bundles), but the disruption in WM integrity may be the core basis for this disconnection of brain regions in schizophrenia^17–20^. At the present, in vivo WM structure is typically assessed by fractional anisotropy (FA) using diffusion tensor imaging (DTI)^21^. FA is a measure of the degree of anisotropy of water molecules diffusion, which is thought to reflect the anatomical features of neural fibers^22,23^. The decrease in FA demonstrates damage of myelin or axons and/or loss coherence^24^. Over the past decades, many studied have reported reduced FA in schizophrenia across widespread brain regions and fiber tracts, suggesting that networks of WM tracts may be changed in schizophrenia^25–28^, which may lead to “disconnection” of the gray matter regions which they link^29,30^. Since WM fiber tracts form the basis of the high-speed communication between brain regions, the alterations of the WM pathways may be implicated in the clinical and cognitive symptoms associated with schizophrenia^31–41^. Several studies found that DTI-measured brain FA was associated with positive symptoms or negative symptoms in chronic medicated patients with schizophrenia^40^. Only a few DTI studies have investigated the WM structure in first-episode and drug-naive (FEDN) patients with schizophrenia^28,42-46^, with mixed results. Further, reports on the relationship between DTI measures and clinical variables in FEDN schizophrenia are sparse.

In the view of sex differences in clinical phenotypes in schizophrenia, and DTI as an important and non-invasive technique to measure WM structure that may be involved in clinical symptoms associated with schizophrenia, it would be of great interest to explore sex differences in FA values in schizophrenia, as well as the association of whole-brain FA alteration with clinical symptoms in schizophrenia, which to our best knowledge, have not been reported yet. We hypothesized that gender differences may exist in FA alteration in multiple brain regions, as well as in association of FA alteration with clinical symptoms in these FEDN patients.

## Materials and methods

### Subjects

We recruited 47 FEDN inpatients from consecutive admission at the initial evaluation for psychosis, and followed for about 3 months as inpatients in order to establish a DSM-IV diagnosis of schizophrenia. The exact follow-up time in average interval was 2.6 ± 0.7 months. All patients met the following inclusion criteria: (1) an acute episode at study intake that met DSM-V criteria for schizophrenia diagnosis, confirmed by two independent experienced psychiatrists based on the Structured Clinical Interview for DSM-IV (SCID); (2) aged 16–45 years, Han Chinese; (3) duration of symptoms not longer than 60 months; (4) no prior treatment with both antipsychotic and non-antipsychotic medications; (5) subjects with sex hormone use history or with some relevant diseases of sex hormone disorder were ruled out. Together, 8 patients were excluded: not meeting the inclusion criteria (*n* = 2), acute clinical status that made interviewing and symptom assessment difficult or unreliable (*n* = 3), and inability to comprehend consent procedures or refusal to sign consent form (*n* = 3). Thus, 39 patients (16 males and 23 females) were included in this study. It is noteworthy that we included a large age range of individuals with FEDN schizophrenia. Among them, 10 patients (25.6%) were under age 20 years, 17 (43.6%) under 30 years and 12 (30.8%) under 45 years. The patients had a mean age of 28.9 ± 10.2 years, a mean duration of illness of 23.4 ± 19.1 months and a mean education of 12.4 ± 3.1 years. In this study, the definition of first episode was the first symptom onset.

Thirty gender- and aged-matched healthy volunteers (13 males and 17 females) were recruited by advertisements at the local community. They had an average age of 27.5 ± 7.9 years and a mean education of 12.3 ± 4.0 years. All healthy controls were interviewed by trained investigators, who were supervised by one of the research psychiatrists. None of them had any personal or family history nor demonstrated any clinical psychiatric disorders by a psychiatric evaluation.

All subjects were Han Chinese recruited at the same period. We obtained a complete medical history and physical examination from all subjects, and any subjects with medical abnormalities were excluded. Neither the patients nor the healthy controls had any history of alcohol or substance dependence (aside from tobacco). All subjects provided signed, informed consent to participate in this study, which was approved by the Institutional Review Board, Beijing, Hui-Long-Guan Hospital, and the First Affiliated Hospital, Shanxi Medical University.

### Clinical assessment

Two psychiatrists who had simultaneously attended a training session in the use of the positive and negative syndrome scale (PANSS). After training, the psychiatrists maintained a correlation coefficient greater than 0.8 for the PANSS total score at repeated assessments. Mean scores on the PANSS were: positive subscore, 25.6 ± 6.0; negative subscale, 18.0 ± 7.3; general psychopathology subscale, 37.8 ± 10.5, and total PANSS Score, 82.4 ± 17.4.

### Imaging acquisition and analysis

DTI data was collected with a 3.0 Tesla General Electric (GE) scanner equipped with an 8-channel brain phased array coil at the Department of Radiology, Peking University First Hospital, Beijing, China. DTI scan was performed with single-shot echo-planar imaging sequence with the following scan parameters: repetition time = 13525 ms, echo time = 77.3 ms, field of view = 256 × 256 mm2, matrix = 128 × 128, 50 slices, thickness = 2.4 mm, skip = 2.4 mm, b-factor = 1000 seconds/mm2, 19 gradient directions, and two averages. The *b* = 0 images were scanned and interspersed three times at the beginning of the acquisition scheme. The patients were scanned within 3 days after they were admitted to hospital, and they remained unmedciated until the scanning.

Voxel-based analysis of the DTI data was carried out using the Functional Magnetic Resonance Imaging of the Brain (FMRIB) software library (FSL; http://www.fmrib.ox.ac.uk/). Firstly, FA images were created by fitting a tensor model to the raw diffusion data using the FMRIB’s Diffusion Toolbox (FDT). Next, brain-extraction was undertaken using the Brain Extraction Tool (BET)^47^. These were referred to as the preprocessing stages. For the next step, all subjects’ FA data were then aligned into a common space using the FMRIB’s Nonlinear Image Registration Tool (FNIRT), which uses a b-spline representation of the registration warp field^30^. Next, the normalized FA image of each subject was resampled to 2 × 2 × 2 mm^3^ Montreal Neurological Institute (MNI) space. This resulted in a standard space version of each FA image. Then a mean FA image was calculated and thinned to generate a mean FA skeleton which represents the centers of all tracts common to the group. A threshold FA value of 0.2 was set^48^. The maximum FA value observed in a direction perpendicular to each tract was assigned to each skeleton voxel. Each subject’s aligned FA data was then projected onto this skeleton and the resulting data were fed into voxel-wise cross-subject statistics^48^. Finally, spatial smoothing was performed with 6 mm full-width half-maximum (FWHM) Gaussian kernel.

### Statistical analysis

Since all variables in Table 1 and Table 2 were normally distributed in patients and normal controls (Kolmogorov–Smirnov one sample test; all *p* > 0.05), all data were presented as mean ± standard deviation (SD) in Table 1 and Table 2, and parametric tests were employed in the following analyses. Group comparisons on demographic and clinical variables used chi squared for categorical variables and analysis of variance (ANOVA) for continuous variables. Group-level analyses were carried out to examine brain regions with significant detectable WM abnormalities in schizophrenia. The voxel-wise FA values were compared between the patient and control subjects using a parametric two-sample *t*-test of Statistical Parametric Mapping 8 (SPM8) software (Wellcome Department of Imaging Neuroscience, London, UK), with gender, age, and education as covariates. Then we included age, education, and smoking as covariates in analyses of covariance (ANCOVA) for gender differences across the brain regions with significant differences in FA values, with independent predictors being gender (male vs. female), diagnosis (patients vs. healthy controls), and the gender-by-diagnosis interaction. Furthermore, among the patient group, ANCOVA was constructed with gender as the independent variable, and the FA values in different brain regions as dependent variables, with age, education, illness course, age of onset, body mass index (BMI), and smoking as the covariates. We assessed relationships between variables with Pearson’s product moment correlation coefficients. We applied Bonferroni corrections to adjust for multiple testing. Lastly, stepwise multiple regression analyses were used to examine the relationships between clinical symptoms shown on PANSS and FA values together with other variables in male and female groups. SPSS version 18.0 was used to do all statistical analysis. All *p* values were 2 tailed at the significance level of < 0.05.

**Table 1:** Demographics and fractional anisotropy (FA) values in FEDN schizophrenia and control subjects

	Schizophrenia		Healthy control				
	Male (*n* = 16)	Female (*n* = 23)_	Male (*n* = 13)	Female (*n* = 17)	Diagnose F (*p* value)	Gender F (*p* value)	Diagnose × Gender F (*p* value)
Age (years)	27.2 ± 10.6	29.2 ± 9.9	27.8 ± 6.3	27.2 ± 9.1	0.26 (0.61)	0.27(0.60)	0.64(0.43)
Education (years)	12.4 ± 3.3	12.5 ± 3.0	13.1 ± 4.1	11.6 ± 3.9	0.001(0.98)	0.62(0.44)	0.91(0.35)
Body mass index (BMI)	24.5 ± 5.2	26.1 ± 4.9	26.2 ± 4.7	25.6 ± 4.3	0.77(0.38)	0.54(0.46)	2.93(0.09)
FA values							
Cerebellum (left)	0.277 ± 0.012	0.277 ± 0.016	0.295 ± 0.012	0.291 ± 0.012	22.37( < .0001)	0.52(0.47)	0.31(0.58)
Brainstem (right)	0.397 ± 0.0134	0.396 ± 0.011	0.417 ± 0.012	0.408 ± 0.012	28.57( < 0.001)	2.96(0.09)	1.66(0.20)
Cingulate (left)	0.435 ± 0.024	0.454 ± 0.028	0.462 ± 0.024	0.474 ± 0.023	13.78( < 0.001)	6.40(0.014)	0.29(0.60)
Internal capsule (right)	0.357 ± 0.015	0.364 ± 0.013	0.373 ± 0.015	0.380 ± 0.010	22.07( < 0.001)	4.83(0.032)	0.004(0.95)
Corpus callosum (right)	0.473 ± 0.024	0.484 ± 0.026	0.499 ± 0.018	0.496 ± 0.021	11.49( < 0.001)	0.41(0.52)	1.47(0.23)

*FA* fractional anisotropy, *FEDN* first-episode and drug-naive

**Table 2:** Clinical characteristics of male and female patients with schizophrenia

	Male	Female	F	*p* value
Age of onset (years)	24.4 ± 6.4	26.3 ± 7.5	1.76	0.12
Duration of illness (years)	2.8 ± 1.9	2.9 ± 1.8	0.01	0.93
Family history of psychosis	3	4	0.06	0.86
Smoking	4	2	1.93	0.17
Body mass index (BMI)	22.0 ± 4.4	21.0 ± 3.3	0.74	0.40
PANSS				
Positive symptom scale	28.7 ± 7.4	22.8 ± 5.3	8.40	0.006
Negative symptom scale	23.3 ± 11.2	18.5 ± 5.5	3.15	0.08
General psychopathology scale	50.5 ± 13.0	36.3 ± 5.8	20.97	0.001
Total score	102.5 ± 25.2	78.1 ± 11.5	16.34	0.001

Mean ± SD

*PANSS* the positive and negative syndrome scale

## Results

### Sample characteristics

Table 1 shows the demographic data of the subjects. We did not find any gender difference in any demographic parameters either for the whole group or when the healthy controls and patients were examined separately (all *p* > 0.05). In addition, we did not find the significant association between age and FA values in all brain regions in either patients or healthy controls, or between age and clinical symptoms in patients.

### FA values in schizophrenia and healthy controls grouped by gender

Compared with the controls, the patients showed widespread areas of lower FA, including corpus callosum, brainstem, internal capsule, cingulate, and cerebellum (all adjusted *p* < 0.01) (Table 1). MANCOVA also revealed overall main effects for gender in FA values in left cingulate (*F* = 6.40, df = 0.014) and in right internal capsule (*F* = 4.83, df = 0.032), with women having higher FA values than men. Further, we examined FA values in these 5 brain regions in males and females separately in the patients and controls. Female patients showed higher FA values in left cingulate than male patients (*F* = 4.92, *p* = 0.033). After controlling for age and education, this difference remained significant (*F* = 5.62, *p* = 0.023). However, it did not pass the Bonferroni correction. In the control group, the males and females showed no significant differences in the FA values in any brain regions (all *p* > 0.05) (Table 1).

### Relationship between FA values and psychopathology in schizophrenia

The males had significantly higher scores than females on the PANSS total, positive and general psychopathology subscale scores (all *p* < 0.01), without significant difference in the PANSS negative subscale score (*p* > 0.05) (Table 2).

**Fig. 1:**
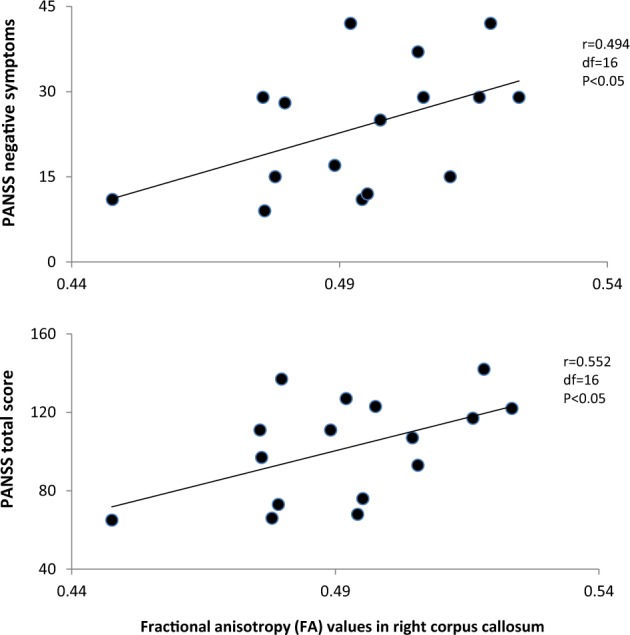
Positive assocations between the fractional anisotropy (FA) values in right corpus callosum and clincial symptoms. For male patients, correlation analysis showed significantly positive associations between the FA values in right corpus callosum and PANSS total score (*r* = 0.552, df = 16, *p* < 0.05) or the negative symptom subscore (*r* = 0.494, df = 16, *p* < 0.05)

For male patients, correlation analysis showed significantly positive associations between the FA values in right corpus callosum and PANSS total score (*r* = 0.552, df = 16, *p* < 0.05; Fig. 1) or the negative symptom subscore (*r* = 0.494, df = 16, *p* < 0.05; Fig. 1). However, these two significances did not pass Bonferroni corrections. Further, multivariate regression analysis showed that FA values in corpus callosum were independently associated with the PANSS negative symptom score (beta = 0.494, *t* = 2.20, *p* = 0.044), and with the PANSS total score (beta = 0.785, *t* = 3.76, *p* = 0.002).

For female patients, correlation analysis showed a trend toward significantly negative association between FA values in left cerebellum and the PANSS positive symptom (*r* = −0.392, df = 22, *p* = 0.07). Multivariate regression analysis showed that FA values in left cerebellum were independently associated with the PANSS positive symptom (beta = −0.717, *t* = −2.25, *p* = 0.041).

## Discussion

We found lower FA values in five brain regions in FEDN schizophrenia patients than healthy controls, suggesting that the FA abnormalities are present even at the early course of first-episode schizophrenia^49^. Our result of widespread low FA in 5 brain regions in FEDN schizophrenia patients is consistent with the majority of the studies evaluating FA values in chronic patients with schizophrenia, as well as in first-episode schizophrenia^20,26,28,32,42,44,50–54^. However, some other studies failed to find any difference in FA values between FEDN patients and healthy controls^55–57^, or even higher FA values in schizophrenia^51^. Several factors may account for these divergent results, for example, differences in DTI techniques including scanner differences in field strengths, head coils and sequence parameters, different filter selection, differences in FA analysis, sampling of patients in different stages of disease progression, different illness courses, or the biological/ethnic heterogeneity.

Further, we found a sex difference in FA values in left cingulate only in the patients, which showed lower FA values in left cingulate in male than female patients, suggesting that female patients could be protected from disrupted connectivity between brain regions of schizophrenia at the acute stage of the illness episode. Numerous studies have shown sexually dimorphic abnormalities in MRI studies in schizophrenia. It is known that gender differences in neuroanatomical features exist in schizophrenia. For example, larger ventricles and smaller overall frontal and temporal lobe volumes were present in male patients than female patients^58^. Male patients showed significant volume reduction in the left temporal lobe compared to healthy controls, without significant difference between female patients and healthy controls^59^. A previous study showed sex differences in superior temporal gyral measures, with significantly greater cortical complexity in inferior frontal regions in male than female schizophrenia patients^60,61^. Further, significant increases in cortical folding were observed in the right superior frontal cortex in male schizophrenia patients compared with male control subjects, but not between female diagnostic groups^60,61^. Also, the sulcogyral pattern of the orbitofrontal cortex was found to be altered in male, but not in female schizophrenia patients^62^. A recent study reported that geometric abnormalities in the anterior corpus callosum in adolescent onset schizophrenia were sexually dimorphic, showing an increase of dispersion toward the anterior left in the corpus callosum in male relative to female patients^63^. Further, they also reported sex-specific changes in the geometry of corpus callosal fibers interconnecting frontal regions in subjects at familial high risk for schizophrenia^64^. Taken together, these MRI studies have demonstrated sex differences in ventricular size and shape, cortical gyrification, WM geometry, and connection abnormalities in schizophrenia. Since our current findings showed sex-specific changes in FA values in left cingulate in first-episode patients, it may provide an indication of sexually dimorphic abnormalities in neurodevelopmental pathology extant before, and independent of neurodegenerative changes occurring after disease onset, possibly reflecting sex differences in early neurodevelopment.

It is meaningful to speculate the underlying mechanisms why lower FA values in left cingulate were found in male than female patients in our present study. Increasing evidence has shown that the organizational and activational influences of gonadal hormones are the important biological factors that determine sexual differentiation of the brain^65^. For example, several reports show that estrogen may cause sex differences in brain morphology, due to its neuroprotective effect and differential concentration of estrogen receptors in sexually dimorphic brain regions^66^. In addition, it was reported that high levels of estrogen can induce an increase of BDNF production and release^67^, which is widely expressed in the adult brain and plays a critical role in the development, regeneration, survival, and maintenance of neuronal function^68^. Therefore, we speculate that sex-specific changes in FA values in left cingulate may be related to neuroprotective effects of estrogen on brain. However, it is worthy of mentioning that we did not find a sex difference in FA values in the brain regions in the healthy controls. At present, it is not clear why there was a significant difference in FA values between males and females only in patients, but not in controls, which deserves further investigation. In addition, FA values were found to be significantly different in five brain regions between patients and healthy controls; however, sex difference in FA values occurred only in left cingulate in patients. We could not provide a reasonable explanation why sex-specific FA change was only present in a specific brain region due to the nature of our cross-sectional design. Future studies with larger samples are needed to confirm this sex difference using a longitudinal design.

Interestingly, we further found gender differences in the relationships between lower FA values and clinical symptoms, showing that FA values in right corpus callosum were positively associated with the PANSS total and the negative symptom scores in male patients, while FA values in left cerebellum were negatively associated with the PANSS positive symptom score in female patients. The gender difference for the association between FA values and clinical symptoms in patients may be explained by sex hormones. In our present study, the male patients had significantly higher scores than females on the PANSS total, positive, and general psychopathology subscale scores. This female advantage in clinical symptoms may also reflect gonadal hormone effects. Estrogen and testosterone may influence clinical symptoms through dopamine and serotonin effects in specific brain regions^69^. For example, estrogen can decrease dopamine concentrations and modulate sensitivities and numbers of dopamine receptors in the striatum and hippocampus^70^. It is generally assumed that the positive symptoms of schizophrenia are associated with hyperactivity of dopaminergic systems, especially in subcortical cortex^71^. Therefore, females may have better clinical symptoms than males, especially in positive symptoms and general psychopathology. Moreover, female patients may show better improvements in symptoms with antipsychotic treatments potentially through normalizing estrogen’s activity in the brain^72^.

On the other hand, previous studies have shown that estrogen may be a neuroprotective agent, playing an important role in sex differences in schizophrenia^66^. Several reports showed that estrogens may have neuroprotective activity through direct antioxidant effects, as well as through estrogen’s receptor-dependent activities, which may cross-talk with other signaling pathways^66^. Taken together, there is sex difference in association of clinical symptoms and disrupted WM connection in our present study, maybe via the sex hormones. Thus, our finding of a negative association between the PANSS positive symptom score and FA values in left cingulate in female patients may be related to high sex hormone levels, which can induce a high FA values and low positive symptoms. On the contrary, without the neuroprotective and neurotrophic effects of sex hormones, the male patients might have lower FA values and higher negative symptoms, resulting in positive association between FA values in right corpus callosum and the PANSS total and the negative symptom scores in male patients. However, we could not provide a reasonable explanation for the findings that only FA values in left cingulate in females, and FA values in right corpus callosum in males were associated with clinical symptoms, but not FA values in other brain regions, which deserves further investigation.

However, it is worthy of mentioning that the association findings between the FA values and PANSS symptom scores in either male or female patients did not pass Bonferroni corrections or only showed a trend toward significance. Hence, these discussions are only speculative; the real relationships between FA values and clinical symptoms deserves further investigation in a large sample using a longitudinal design.

There are innate limitations to this study. First, the cross-sectional design we used may prevent asserting valid conclusions regarding the sex difference in association of the regional disconnectivity of the brain with clinical symptoms in schizophrenia. A future longitudinal study could likely better reveal this sex difference. Second, our sample size was still relatively small, especially after dividing into two sex groups, due to recruitment difficulties of first-episode and drug-naive patients with schizophrenia. A replication study in an independent sample is needed with a potentially larger sample size and from different ethnic populations in order to test for a false-positive association. Third, although we speculate that the gender difference in association between lowered FA values and clinical symptoms in schizophrenia patients may be due to different sex hormone levels, we did not measure sex hormone levels. Thus, lack of measurement for sex hormones is considered as one of the methodological limitations. Fourth, the age range for the patients was 16–45 years, which is a rather wide age range. Age is one of the influential factors of brain structure. Although covarying for age might control for age effects, the exact influence of age on the DTI results warrant further investigation. Fifth, in our present study, the DTI data were analyzed using voxel-based morphometry, which for DTI, is a substandard approach. Moreover, the voxel-based approach has an advantage in terms of objectivity and no intentional measurements. However, it could potentially be contaminated with registration or normalization errors^62^. Furthermore, using voxel-based morphometry is not the most current approach for DTI^73^. Tract-based spatial statistics (TBSS)^74^ may produce more rigorous results, and will be utilized in our ongoing diffusion tensor magnetic resonance imaging study in a large sample of FES patients. Sixth, although some related factors were adjusted for in the main analyses, many other important factors associated with FA values and clinical symptoms were missing. These factors are especially important as a number of them are at more severe levels in first-episode, untreated patients e.g., stress, anxiety, depression, sleep disruptions, etc. Unfortunately, we did not collect these factors in our present study.
